# Green Approach—Multicomponent Production of Boron—Containing Hantzsch and Biginelli Esters

**DOI:** 10.3390/ijms14022903

**Published:** 2013-01-30

**Authors:** Joel Martínez, Stephany Romero-Vega, Rita Abeja-Cruz, Cecilio Álvarez-Toledano, René Miranda

**Affiliations:** 1 Department of Chemistry, Faculty of Superior Studies Cuautitlan, Field 1, Autonomous National University of Mexico, Cuautitlan Izcalli, State of Mexico, 54740, Mexico; E-Mails: atlanta126@gmail.com (J.M.); goddess_10fany@hotmail.com (S.R.-V.); rita_abeja_33@yahoo.com.mx (R.A.-C.); 2 Institute of Chemistry, Autonomous National University of Mexico, University City, Coyoacan, D.F., 04510, Mexico; E-Mail: cecilio@servidor.unam.mx

**Keywords:** multicomponent reaction, green chemistry, twelve principles, boron-containing-Hantzsch and Biginelli esters, microwave and infrared irradiation, matrix-ion interaction, FAB^+^MS

## Abstract

Multicomponent reactions are excellent methods that meet the requirements of green chemistry, by reducing the number of steps, and consequently reducing purification requirements. Accordingly, in this work, 11 novel hybrid-boron-containing molecules, namely eight 1,4-dihydropyridines and three 3,4-dihydropyrimidinones, derived from formylphenylboronic acids (*ortho*, *meta* and *para*), were obtained using a green approach, involving H-4CR and B-3CR practices, in the presence of ethanol, which is a green solvent, and using three comparatively different modes of activation (mantle heating, yield 3%–7% in 24 h, Infrared Radiation (IR) irradiation, yield 12%–17% in 12 h, and microwave irradiation, yield 18%–80%, requiring very low reaction times of 0.25–0.33 h). In addition, as a green-approach is offered, a convenient analysis, of the 12 green chemistry principles for the overall procedure was performed. Finally, since all the products are new, characterizations were carried out using common analytic procedures (^1^H, ^11^B, and ^13^C NMR, FAB^+^MS, HRMS, and IR). The accurate mass data of unexpected ions related to interactions between thioglycerol and the expected products, in the FAB^+^-mode, enabled unequivocal characterization of the target molecules.

## 1. Introduction

The design and implementation of new sustainable synthetic processes is one of the major challenges in modern organic synthesis. Multicomponent reactions (MCR) are an important subclass of tandem reactions. These reactions are suitable for green organic syntheses because they involve processes in which three or more components react directly to form a unique product, giving good atom economy [1–4]. It is important to note that in such reactions, the isolation of intermediate, products is unnecessary, making the method more sustainable. Hence, the MCRs, also known as “one-pot” processes, are valuable in green organic synthesis because they have many important attributes: A small number of steps, and consequently a simple purification procedure, which enhances the synthetic efficiency, and savings in time, solvents, and other resources. These features are in agreement with following Principles of Green Chemistry [5]: *prevention* (principle 1), the formation of by-products is minimum; *atom economy* (principle 2), the target methodology is designed to maximize the incorporation of the atoms in the starting materials, into the final product; *safer solvents and auxiliaries* (principle 5), it is unnecessary to use solvents for intermediate purification, avoiding the generation of waste; *design for energy efficiency* (principle 6), the use of alternative modes of activation of a particular reaction e.g., microwave or infrared irradiation, instead of typical thermal procedures (mantle heating), significantly reduces the corresponding reaction times, consequently decreasing energy consumption; *reduce derivatization* (principle 8), unnecessary derivatization is avoided (the use of blocking groups, protection/deprotection, temporary modification of physical/chemical processes); *catalytic processes* (principle 9), in many cases eco-friendly catalysts are used; and some others, depending on the specific case.

It is well known that Hantzsch esters (1,4-dihydropyridines) and Biginelli esters (3,4-dihydropirimidinones), produced respectively by the H-4CR and B-3CR procedures, are important classes of calcium channel blockers that have emerged as important drugs for the treatment of cardiovascular diseases, including hypertension, and have many other interesting activities [6–11]. The increasing importance of boron-containing compounds (BCCs) [12,13] supports the development of new BCC-derivatives. Many of these compounds have been used as antiseptics, antibiotics, cosmetics, and insecticides for more than a century; some BCCs have been used to target bio-molecules involved in cancer therapy, including non-specific applications such as boron neutron capture therapy and direct selective action on some cancer targets [14].

**Scheme 1:**
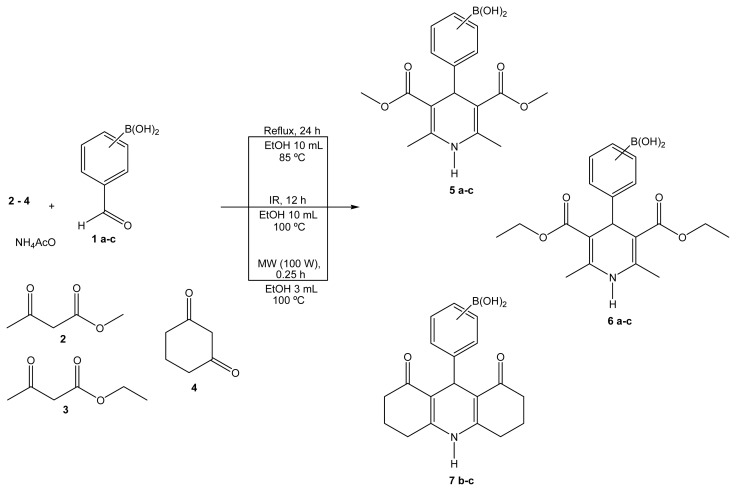
BCDHPy esters production.

**Scheme 2:**
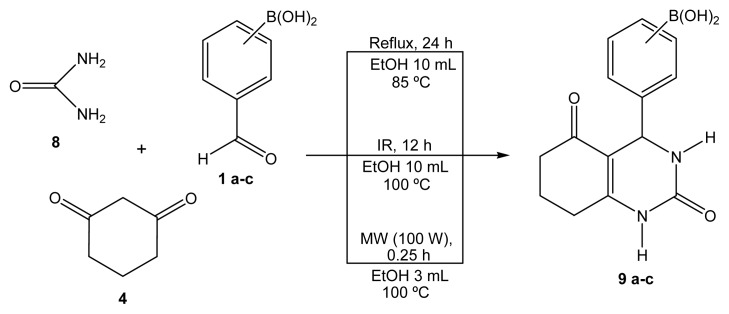
BCDHPm esters production.

As part of our ongoing research program, we are interested in the green production of novel hybrid heterocyclic molecules in order to synergize or modify the pharmacological activities of the prototypes, mainly using microwave or near-infrared irradiation as the activating source, in the absence of solvents and using naturally occurring non-toxic catalysts, such as bentonitic clays [15–19]. The purpose of this work is to investigate the one-pot green production of various hybrid-BCCs related to a set of eight novel 1,4-dihydropyridines **5a**–**c**, **6a**–**c**, **7b**,**c** (BCDHPy; Scheme 1) and to a set of three 3,4-dihydropyrimidinones **9a**–**c** (BCDHPm, Scheme 2), all derivatives of *o*, *m*, or *p*-boronic acids, using three different modes of activation (mantle heating, microwave irradiation, or near-infrared irradiation), in the presence of ethanol, which is a green solvent according to the TRI-EPA (Toxics Release Inventory-Environmental Protection Agency) [20]. Finally, considering that the 11 molecules are new, appropriate spectroscopic characterizations were performed. The molecular masses of the target products were determined using certain ions, identified by FAB^+^-HRMS (fast atom bombardment-high resolution mass spectroscopy) using thioglycerol as the matrix. We are convinced that any novel green-contribution related to BCC-hybrids with promising biological activities, and to H-4CR and B-3CR procedures. It is worth mentioning that the molecules obtained in the present work are under study as vasodilators and apoptotic inducers.

## 2. Results and Discussion

### 2.1. Synthesis

MCRs are appropriate protocols for assembling different heterocyclic moieties. As part of our ongoing research program on the production of biologically active heterocyclic molecules using suitable green approaches, using mainly novel modes to activate reactions [15–19], we report, in this article, the development of 11 new boron-containing heterocyclic molecules. The first eight compounds **5a**–**c**, **6a**–**c** and **7b**,**c** were prepared by adapting the H-4CR protocol, using regioisomers (*ortho*, *meta*, and *para*) of formylphenylboronic acid, three different β-dicarbonylic compounds, and ammonium acetate, using-comparatively mantle heating, and infrared and microwave irradiation as the activating modes, with a minimal quantity of ethanol (Scheme 1). The complementary target molecules **9a**–**c** were obtained by a B-3CR protocol using the regioisomers (*ortho*, *meta*, and *para*) of formylphenylboronic acid with 1,3-cyclohexanedione in the presence of urea and a minimal quantity of ethanol (Scheme 2), again using mantle heating, and infrared and microwave irradiation as the activating modes. Interestingly, the reactions proceeded using formylphenylboronic acids both as reagent and as catalyst.

**Scheme 3:**
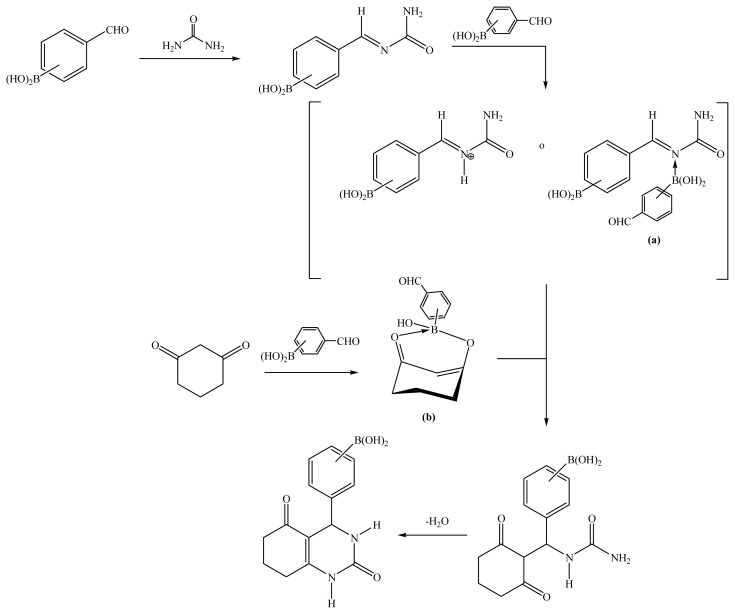
Interaction between formylphenylboronic acids and urea or 1,3-ciclohexanedione.

As can be seen in Table 1, the best yields of the target molecules (**7a** was no formed, and low yield of **6a**, possibly as result of steric hindrance), all of which are new, were obtained by microwave irradiation; lower yields were obtained using typical mantle heating or infrared irradiation. Very short reaction times were required using microwave irradiation (only a few minutes, *versus* many hours for mantle heating and infrared irradiation). Microwave irradiation is directly absorbed by the solvent and the reagents, resulting in a rapid temperature rise in the system, and increasing the reactivity [21]; this fits the sixth principle of the green chemistry, *i.e.*, decreasing energy consumption. Also, ethanol, used as the solvent, has a high tan δ value (0.941) that is the ability of this substance to convert electromagnetic energy into heat [22], also favoring efficient energy absorption. Furthermore, ethanol is also considered to be a green solvent because of its low toxicity and good degradability [20,23], in accordance with the fifth principle of the green protocol. Formylphenylboronic acids are also considered to be green compounds because of their low toxicities [24,25] and ultimate degradation in the environment (to eco-friendly boric acid), in accordance with the third and twelfth green chemistry principles. In the production of the BCDHPy esters, ammonium acetate generates acetic acid, which is classified as non-toxic (TRI-EPA), in accordance with the third and twelfth principles. In the production of the BCDHPm esters, urea does not reach its decomposition temperature (160 °C), therefore toxic by-products (biuret, carbon dioxide, and cyanuric acid), are not generated, supporting the first, third, and twelfth green chemistry principles. Finally, with regard to the moderate yields of the BCDHPm esters, it can be assumed that an interaction occurs between formylphenylboronic acid and urea (a) or 1,3-ciclohexanedione (b), either by protonation or coordination with the boronic moiety, Scheme 3, according to Debache *et al.* [26].

### 2.2. Spectroscopic Characterization

**Figure 1:**
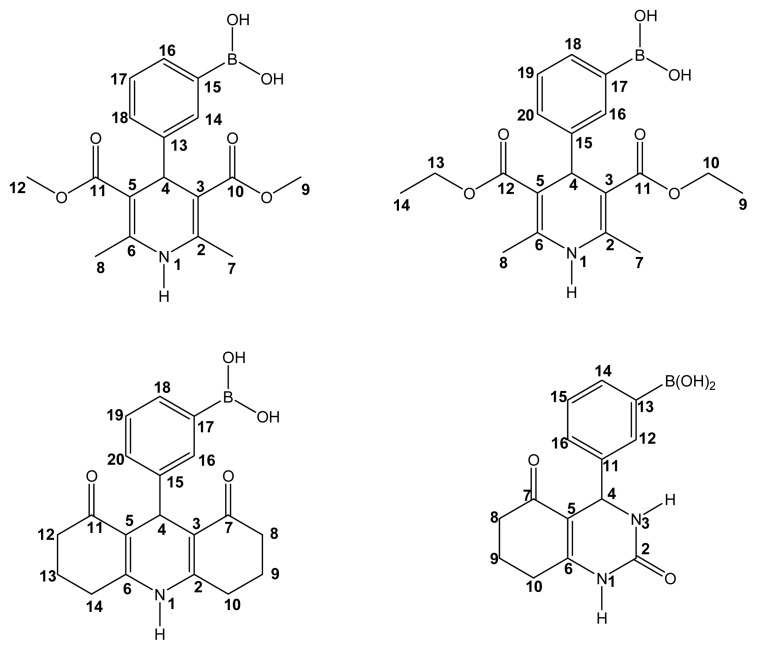
Positions of BCDHPy and BCDHPm esters.

The nomenclature used is that for Hantzsch (1,4-dihydropyridine) and Biginelli (3,4-dihydropyrimidinone) esters systems, and positions are assigned according to the examples shown in Figure 1.

#### Boron-containing-Hantzsch esters

The ^1^H NMR spectra of the eight BCDHPy esters exhibited the expected singlet for the benzylic hydrogen (H4) between 4.83 and 5.02 ppm; another singlet, between 8.82 and 9.57 ppm, was unequivocally assigned to the proton attached to the nitrogen of the dihydropyridine ring; in addition a single signal (integrating two hydrogens) between 7.60 and 8.38 ppm was assigned to the hydroxyl groups of the boronic residue. The absence of signals between 10.02 and 10.18 ppm indicated the disappearance of the hydrogen of the aldehyde groups of reagents **1a**–**c**. The expected ^1^H NMR patterns for the ethyl, methyl, methylene, and aryl functions were also observed as show in the experimental data for **5a**–**c**, **6a**–**c**, and **7b**,**c**. In the ^13^C NMR spectra, the signal between 36.7 and 38.9 ppm was assigned to the benzylic carbon (C4); it is worth noting that the signal corresponding to the carbon *ipso* to the boron atom was not observed, maybe as a result of a quadrupole effect, and, in addition, the absence of a signal between 193.6 and 194.4 ppm clearly indicated the disappearance of the aldehyde groups of **1a**–**c**. In the ^11^B NMR spectra, compounds **5b**,**c**, **6a**–**c**, and **7b**,**c** showed signals assigned to trivalent boron at 33.88, 35.39, 37.31, 34.04, 35.56, 37.54, 34.74, and 37.60 ppm, respectively. In contrast, **5a**, and **6a**,**b**, exhibited signals at −6.93, 8.56, and 1.03 ppm, consistent with tetravalent boron. The respective IR spectra exhibited interesting bands between 1336 and 1373 cm^−1^ arising from B–O and a band from B–C between 1018 and 1045 cm^−1^ demonstrating the presence of the boronic moiety in all these compounds. In addition, the expected vibrations arising from carbonyl, hydroxyl, and double bond functions were seen, as shown in the corresponding experimental data.

#### Boron-containing-Biginelli esters

The ^1^H NMR spectra of the three BCDHPm esters exhibited the expected signal for the benzylic hydrogen (H4) between 5.43 and 6.48 ppm, another signal between 8.27 and 8.79 ppm was unequivocally assigned to the hydrogen attached to the nitrogen (N-1), and the signal between 6.93 and 8.31 was assigned to the proton attached to the other nitrogen (N-3). In addition, a singlet (integrating two hydrogens) between 7.21 and 8.57 was assigned to the hydroxyl groups of the boronic residues. The absence of a signal between 10.02 and 10.18 ppm indicated the disappearance of the hydrogens of the aldehyde groups of reagents **1a**–**c**. The expected ^1^H NMR patterns for methylene and aryl functions were also observed, as show, in the corresponding experimental data for **9a**–**c**. In the ^13^C NMR spectra, the signal at 36.4 ppm was assigned to the benzylic carbon; it is worth nothing that the signal corresponding to the carbon *ipso* to the boron atom was not observed, maybe as a result of a quadrupole effect. In addition, the absence of a signal between 193.6 and 194.4 ppm clearly indicated the disappearance of the aldehyde groups of **1a**–**c**. In the ^11^B NMR spectra, all the compounds showed signals assigned trivalent boron at 29.26, 32.89, and 38.92 ppm. However, **9a** exhibited a signal at 1.18 ppm, corresponding to tetravalent boron. The respective IR spectra exhibited bands between 1368 and 1390 cm^−1^ arising from B–O and a band from B–C between 1021 and 1062 cm^−1^ demonstrating the presence of a boronic moiety in all these compounds. In addition, the expected vibrations arising from carbonyl, hydroxyl, and double bond functions were seen, as show in the corresponding experimental data.

#### Mass spectral data for BCDHPy and BCDHPm

The expected molecular ions were not observed in the three different ionization modes (electron impact (EI), chemical ionization (CI) and fast atom bombardment (FAB^+^) with glycerol as the matrix); however, it is important to note that the mass spectra were obtained using thioglycerol as the matrix, and interesting ions known as artifacts (previously referred to as *quasi*-molecular ions) were observed during the FAB^+^ analysis. These ions, related to the molecular-ions, were recognized and subjected to high-resolution experiments, thus their accurate mass data and consequently their unequivocal elemental compositions were obtained [27].

## 3. Experimental Section

### 3.1. General

Reagents and solvents used were purchased from Aldrich Chemical and Merck and were used without further treatment. The reactions were monitored by thin layer chromatography (TLC) (*n*-hexane/EtOAc, 6:4) and visualization was achieved using a 254 nm UV lamp. NMR experiments were conducted using a Varian Mercury-200 spectrometer at 200 MHz and 50 MHz for hydrogen and carbon, respectively. An Oxford-300 spectrometer at 96 MHz was used for the boron experiments; the solvent was DMSO-*d*_6_, and multiplicities are reported as singlet (s), doublet (d), triplet (t), quartet (q) and multiplet (m). The FAB^+^MS and HRMS measurements were determined using a JEOL JMS-700 MStation mass spectrometer with thioglycerol as the matrix. The IR spectra were acquired using a Perkin-Elmer spectrometer (KBr disks). The melting points were determined on a Fisher-Johns apparatus and are uncorrected. The microwave-assisted production of the target compounds was performed using a CEM Focused Microwave™ Synthesis System; the infrared irradiation was generated using a Philips IR lamp (375 W/220 V), as described in a previous publication by our research group [28]. Conventional thermal heating was performed using a heating mantle.

### 3.2. General Synthesis

#### Boron-containing-Hantzsch esters

In an appropriate micro scale glass vessel, 1.000 mmol (150 mg) of **1a**, **1b** or **1c**, 1.980 mmol (230 mg) of methyl acetoacetate (**2**), and 1.999 mmol (260 mg) of ethyl acetoacetate (**3**) or 1.962 mmol (220 mg) of 1,3-cyclohexanedione (**4**) together with 9.989 mmol (770 mg) of NH_4_AcO were mixed. The mixtures were treated using different activation modes: Microwave irradiation for 15 min at 100 °C with 3 mL of ethanol, infrared irradiation for 12 h at 100 °C, on a sealed vessel, with 10 mL of ethanol, and under thermal conditions for a period of 24 h under reflux with 10 mL ethanol. The reaction progress was monitored by TLC (silica gel/*n*-hexane/ethyl acetate, 6:4); the reaction mixture was washed using 15 mL of cold water and then 5 mL of hot ethanol, giving pure products (Table 1).

##### 2-(3,5-Bis(methoxycarbonyl)-2,6-dimethyl-1,4-dihydropyridin-4-yl)phenylboronic acid (**5a**)

56% yield; mp 190–195 °C; ^1^H NMR (DMSO-*d*_6_) δ: 9.03 (s, 1H, NH), 8.20 (s, 2H, OH), 7.34–6.99 (m, 4H, H-Ar), 5.01 (s, 1H, H-4), 3.53 (s, 6H, OMe), 2.26 (s, 6H, H-7 and 8); ^13^C NMR (DMSO-*d*_6_) δ 168.7 (C-10 and 11, C=O), 152.5 (C-13, C-Ar), 145.9 (C-2 and 6, C=C), C-14 no observed, carbon *ipso* to boron, 132.3 (C-15, C-Ar), 129.9 (C-18, C-Ar), 128.3 (C-16, C-Ar), 126.0 (C-17, C-Ar), 103.1 (C-3 and 5, C=C), 51.2 (C-9 and 12, OCH_3_), 38.9 (C-4, CH), 18.8 (C-7 and 8, CH_3_); ^11^B (DMSO-*d*_6_) δ: −6.93 as tetravalent boron; IR (KBr) cm^−1^: 3390 (OH), 1666 (C=O), 1609 (C=C), 1366 (B-O), 1022 (B-C); FAB^+^*m*/*z* (% ra) (6 KeV): 416(17) ion-matrix interaction [M + 71]^+^, 224(100) [M − 121]^+^; FAB^+^-HRMS: Molecular-Matrix interaction ion, C_20_H_23_^11^B_1_N_1_O_6_S_1_, observed 416.1334 Da/estimated 416.1339 Da, base peak C_11_H_14_N_1_O_4_ observed 224.0917 Da/estimated 224.0917 Da; isotopic contribution (^1^B) *versus* the loss of hydrogen, C_20_H_23_^1^B_1_N_1_O_6_S_1_, observed 415.1349 Da/estimated 415.1375 Da, and for C_20_H_22_^11^B_1_N_1_O_6_S_1_, observed 415.1255 Da/estimated 415.1261 Da.

##### 3-(3,5-Bis(methoxycarbonyl)-2,6-dimethyl-1,4-dihydropyridin-4-yl)phenylboronic acid (**5b**)

72% yield; mp 155–158 °C; ^1^H NMR (DMSO-*d*_6_) δ: 8.88 (s, 1H, NH), 7.95 (s, 2H, OH), 7.57–6.83 (m, 4H, H-Ar), 4.88 (s, 1H, H-4), 3.53 (s, 6H, OMe), 2.25 (s, 6H, H-7 and 8); ^13^C NMR (DMSO-*d*_6_) δ: 167.5 (C-10 and 11, C=O), 146.8 (C-13, C-Ar), 145.6 (C-2 and 6, C=C), C-15 no observed, carbon *ipso* to boron, 133.1 (C-14 and 16, C-Ar), 129.0 (C-17 and 18, C-Ar), 101.7 (C-3 and 5, C=C), 50.7 (C-9 and 12, OCH_3_), C-4 is no observed (CH), 18.3 (C-7 and 8, CH_3_); ^11^B (DMSO-*d*_6_) δ: 33.88 as trivalent boron; IR (KBr) cm^−1^: 3327 (OH), 1680 (C=O), 1598 (C=C), 1336 (B-O), 1022 (B-C); FAB^+^*m*/*z* (% ra) (6 KeV): 416(10) ion-matrix interaction [M + 71]^+^, 224(100) [M − 121]^+^; FAB^+^-HRMS: Molecular matrix interaction ion, C_20_H_23_^11^B_1_N_1_O_6_S_1_, observed 416.1334 Da/estimated 416.1339 Da, base peak C_11_H_14_N_1_O_4_, observed 224.0917 Da/estimated 224.0917 Da; isotopic contribution (^1^B) *versus* the loss of hydrogen, for C_20_H_23_^1^B_1_N_1_O_6_S_1_, observed 415.1349 Da/estimated 415.1375 Da, for C_20_H_22_^11^B_1_N_1_O_6_S_1_, observed 415.1255 Da/estimated 415.1261 Da.

##### 4-(3,5-Bis(methoxycarbonyl)-2,6-dimethyl-1,4-dihydropyridin-4-yl)phenylboronic acid (**5c**)

80% yield; mp 144–146 °C; ^1^H NMR (DMSO-*d*_6_) δ: 8.96 (s, 1H, NH), 7.93 (s, 2H, OH), 7.61 (d, 2H, H-15 and 17, H-Ar), 7.09 (d, 2H, H-14 and 18, H-Ar), 4.88 (s, 1H, H-4), 3.54 (s, 6H, OMe), 2.26 (s, 6H, H-7 and 8); ^13^C NMR (DMSO-*d*_6_) δ: 167.4 (C-10 and 11, C=O), 149.6 (C-13, C-Ar), 145.8 (C-2 and 6, C=C), C-16 no observed, carbon *ipso* to boron, 134.1 (C-15 and 17, C-Ar), 126.1 (C-14 and 18, C-Ar), 101.4 (C-3 and 5, C=C), 50.7 (C-9 and 12, OCH_3_), 38.2 (C-4, CH), 18.4 (C-7 and 8, CH_3_); ^11^B (DMSO-*d*_6_) δ: 35.39 as trivalent boron; IR (KBr) cm^−1^: 3301 (OH), 1664 (C=O), 1605 (C=C), 1340 (B-O), 1019 (B-C); FAB^+^*m*/*z* (% ra) (6 KeV): 416(7) ion-matrix interaction [M + 71]^+^, 224(100) [M − 121]^+^; FAB^+^-HRMS: Molecular matrix interaction ion, C_20_H_23_^11^B_1_N_1_O_6_S_1_, observed 416.1334 Da/estimated 416.1339 Da, base peak for C_11_H_14_N_1_O_4_, observed 224.0917 Da/estimated 224.0917 Da; isotopic contribution (^1^B) *versus* the loss of hydrogen for C_20_H_23_^1^B_1_N_1_O_6_S_1_, observed 415.1349 Da/estimated 415.1375 Da, for C_20_H_22_^11^B_1_N_1_O_6_S_1_, observed 415.1255 Da/estimated 415.1261 Da.

##### 2-(3,5-Bis(ethoxycarbonyl)-2,6-dimethyl-1,4-dihydropyridin-4-yl)phenylboronic acid (**6a**)

31% yield; mp 156–160 °C; ^1^H NMR (DMSO-*d*_6_) δ: 8.97 (s, 1H, NH), 8.27 (s, 2H, OH), 7.33–7.05 (m, 4H, H-Ar), 5.02 (s, 1H, H-4), 4.01 (q, 4H, H-10 and 13), 2.26 (s, 6H, H-7 and 8), 1.15 (t, 6H, H-9 and 14); ^13^C NMR (DMSO-*d*_6_) δ: 168.3 (C-11 and 12, C=O), 152.8 (C-15, C-Ar), 145.7 (C-2 and 6, C=C), C-16 no observed, carbon *ipso* to boron, 129.8 (C-17, C-Ar), 128.2 (C-20, C-Ar), 126.3 (C-18, C-Ar), 125.2 (C-19, C-Ar), 103.3 (C-3 and 5, C=C), 59.8 (C-10 and 13, OCH_2_), C-4 no observed (CH), 18.9 (C-7 and 8, CH_3_), 14.3 (C-9 and 14, OCH_2_CH_3_); ^11^B (DMSO-*d*_6_) δ: 37.31 as trivalent boron and 8.56 as tetravalent boron; IR (KBr) cm^−1^: 3326 (OH), 1663 (C=O), 1608 (C=C), 1368 (B-O), 1022 (B-C); FAB^+^*m*/*z* (% ra) (6 KeV): 444(8) ion-matrix interaction [M + 71]^+^, 252(100) [M − 121]^+^; FAB^+^-HRMS: Molecular matrix interaction ion, for C_22_H_27_^11^B_1_N_1_O_6_S_1_, observed 444.1647 Da/estimated 444.1652 Da, base peak C_13_H_18_N_1_O_4_, observed 252.1230 Da/estimated 252.1236 Da; isotopic contribution (^1^B) *versus* the loss of hydrogen for C_22_H_27_^1^B_1_N_1_O_6_S_1_, observed 443.1685 Da/estimated 443.1688 Da, for C_22_H_26_^11^B_1_N_1_O_6_S_1_, observed 443.1568 Da/estimated 443.1574 Da.

##### 3-(3,5-Bis(ethoxycarbonyl)-2,6-dimethyl-1,4-dihydropyridin-4-yl)phenylboronic acid (**6b**)

72% yield; mp 198–201 °C; ^1^H NMR (DMSO-*d*_6_) δ: 8.83 (s, 1H, NH), 7.63–7.12 (m, 4H, H-Ar), 7.60 (s, 2H, OH), 4.84 (s, 1H, H-4), 3.96 (q, 4H, H-10 and 13), 2.24 (s, 6H, H-7 and 8), 1.21 (t, 6H, H-9 and 14); ^13^C NMR (DMSO-*d*_6_) δ: 167.1 (C-11 and 12, C=O), 147.1 (C-15, C-Ar), 145.2 (C-2 and 6, C=C), C-17 no observed, carbon *ipso* to boron, 133.8 (C-16, C-Ar), 132.0 (C-18, C-Ar), 129.6 (C-20, C-Ar), 127.7 (C-19, C-Ar), 102.1 (C-3 and 5, C=C), 59.0 (C-10 and 13, OCH_2_), C-4 no observed (CH), 18.4 (C-7 and 8, CH_3_), 14.2 (C-9 and 14, OCH_2_CH_3_); ^11^B (DMSO-*d*_6_) δ: 34.04 as trivalent boron and 1.03 as tetravalent boron; IR (KBr) cm^−1^: 3323 (OH), 1680 (C=O), 1603 (C=C), 1373 (B-O), 1021 (B-C); FAB^+^*m*/*z* (% ra) (6 KeV): 444(12) ion-matrix interaction [M + 71]^+^, 252(100) [M − 121]^+^; FAB^+^-HRMS: Molecular matrix interaction ion, for C_22_H_27_^11^B_1_N_1_O_6_S_1_, observed 444.1647 Da/estimated 444.1652 Da, base peak C_13_H_18_N_1_O_4_, observed 252.1230 Da/estimated 252.1236 Da; isotopic contribution (^1^B) *versus* the loss of hydrogen, for C_22_H_27_^1^B_1_N_1_O_6_S_1_, observed 443.1685 Da to 443.1688 Da, both values for C_22_H_26_B_1_N_1_O_6_S_1_ is 443.1568 Da to 443.1574 Da.

##### 4-(3,5-Bis(ethoxycarbonyl)-2,6-dimethyl-1,4-dihydropyridin-4-yl)phenylboronic acid (**6c**)

69% yield; mp 150–154 °C; ^1^H NMR (DMSO-*d*_6_) δ: 8.82 (s, 1H, NH), 8.19 (s, 2H, OH), 7.99 (d, 2H, H-17 and 19, H-Ar), 7.61 (d, 2H, H-16 and 20, H-Ar), 4.86 (s, 1H, H-4), 3.98 (q, 4H, H-10 and 13), 2.26 (s, 6H, H-7 and 8), 1.00 (t, 6H, H-9 and 14); ^13^C NMR (DMSO-*d*_6_) δ: 167.0 (C-11 and 12, C=O), 145.4 (C-15, C-Ar), 142.2 (C-2 and 6, C=C), C-18 no observed, carbon *ipso* to boron, 134.0 (C-17 and 19, C-Ar), 126.9 (C-16 and 20, C-Ar), 101.8 (C-3 and 5, C=C), 59.1 (C-10 and 13, OCH_2_), C-4 no observed (CH), 18.4 (C-7 and 8, CH_3_), 14.3 (C-9 and 14, OCH_2_CH_3_); ^11^B (DMSO-*d*_6_) δ: 35.56 as trivalent boron; IR (KBr) cm^−1^: 3322 (OH), 1681 (C=O), 1608 (C=C), 1372 (B-O), 1020 (B-C); FAB^+^*m*/*z* (% ra) (6 KeV): 444(13) ion-matrix interaction [M + 71]^+^, 252(100) [M − 121]^+^; FAB^+^-HRMS: Molecular matrix interaction ion, for C_22_H_27_^11^B_1_N_1_O_6_S_1_, observed 444.1647 Da/estimated 444.1652 Da, base peak C_13_H_18_N_1_O_4_, observed 252.1230 Da/estimated 252.1236 Da; isotopic contribution (^1^B) *versus* the loss of hydrogen, for C_22_H_27_^1^B_1_N_1_O_6_S_1_, observed 443.1685 Da/estimated 443.1688 Da, for C_22_H_26_^11^B_1_N_1_O_6_S_1_, observed 443.1568 Da/estimated 443.1574 Da.

##### 3-(1,1′-Dioxociclohex-2-en[3,2-b,2′,3′-e]-1,4-dihydropyridin-4-yl)phenylboronic acid (**7b**)

60% yield; mp >300 °C; ^1^H NMR (DMSO-*d*_6_) δ: 9.53 (s, 1H, NH), 8.38 (s, 2H, OH), 7.54–7.10 (m, 4H, H-Ar), 4.89 (s, 1H, H-4), 2.16-1.76 (m, 12H, H-8-10 and 12-14); ^13^C NMR (DMSO-*d*_6_) δ: 194.8 (C-7 and 11, C=O), 151.3 (C-15, C-Ar), 146.3 (C-2 and 6, C=C), C-17 no observed, carbon *ipso* to boron, 133.5 (C-16, C-Ar), 131.5 (C-18, C-Ar), 129.7 (C-20, C-Ar), 126.8 (C-19, C-Ar), 112.7 (C-3 and 5, C=C), 36.9 (C-4, CH), 32.8 (C-8 and 12, CH_2_), 26.4 (C-10 and 14, CH_2_), 20.9 (C-9 and 13, CH_2_); ^11^B (DMSO-*d*_6_) δ: 34.74 as trivalent boron; IR (KBr) cm^−1^: 3384 (OH), 1614 (C=O), 1366 (B-O), 1045 (B-C); FAB^+^*m*/*z* (% ra) (6 KeV): 410(13) ion-matrix interaction [M + 90 − 17]^+^, 216(100) [M − 121]^+^; FAB^+^-HRMS: Molecular matrix interaction ion, for C_22_H_25_^11^B_1_N_1_O_4_S_1_, observed 410.1592 Da/estimated 410.1597 Da, base peak C_13_H_14_N_1_O_2_, observed 216.1019 Da/estimated 216.1025 Da; isotopic contribution (^1^B) *versus* the loss of hydrogen, for C_22_H_25_^1^B_1_N_1_O_4_S_1_, observed 409.1626 Da/estimated 409.1634 Da, for C_22_H_24_^11^B_1_N_1_O_4_S_1_, observed 409.1514 Da/estimated 409.1519 Da.

##### 4-(1,1′-Dioxociclohex-2-en[3,2-b,2′,3′-e]-1,4-dihydropyridin-4-yl)phenylboronic acid (**7c**)

43% yield; mp >300 °C; ^1^H NMR (DMSO-*d*_6_) δ: 9.57 (s, 1H, NH), 7.92 (s, 2H, OH), 7.58–7.03 (m, 4H, H-Ar), 4.90 (s, 1H, H-4), 2.19–1.76 (m, 12H, H-8-10 and 12-14); ^13^C NMR (DMSO-*d*_6_) δ: 194.9 (C-7 and 11, C=O), 151.4 (C-15, C-Ar), 147.4 (C-2 and 6, C=C), C-18 no observed, carbon *ipso* to boron, 133.9 (C-17 and 19, C-Ar), 127.8 (C-16 and 20, C-Ar), 112.4 (C-3 and 5, C=C), 36.8 (C-4, CH), 32.0 (C-8 and 12, CH_2_), 26.3 (C-10 and 14, CH_2_), 20.8 (C-9 and 13, CH_2_); ^11^B (DMSO-*d*_6_) δ: 37.60 as trivalent boron; IR (KBr) cm^−1^: 3273 (OH), 1614 (C=O), 1365 (B-O), 1018 (B-C); FAB^+^*m*/*z* (% ra) (6 KeV): 410(7) ion-matrix interaction [M + 90 − 17]^+^, 216(100) [M − 121]^+^; FAB^+^-HRMS: Molecular matrix interaction ion, for C_22_H_25_^11^B_1_N_1_O_4_S_1_, observed 410.1592 Da/estimated 410.1597 Da, base peak, for C_13_H_14_N_1_O_2_, observed 216.1019 Da/estimated 216.1025 Da; isotopic contribution (^1^B) *versus* the loss of hydrogen, for C_22_H_25_^1^B_1_N_1_O_4_S_1_, observed 409.1626 Da/estimated 409.1634 Da, for C_22_H_24_^11^B_1_N_1_O_4_S_1_ observed 409.1514 Da/estimated 409.1519 Da.

#### Boron-containing-Biginelli esters

In a suitable glass vessel, 1.000 mmol (150 mg) of **1a**, **1b** or **1c**, 0.981 mmol (110 mg) of 1,3-cyclohexanedione (**4**) and 9.990 mmol (600 mg) of urea (**8**) were placed. The mixtures were irradiated with microwaves for 20 min at 140 °C with 3 mL of ethanol, with infrared irradiation for 12 h at 100 °C, on a sealed vessel, with 10 mL of ethanol, and under thermal conditions for 24 h under reflux with 10 mL of ethanol; the reaction progress was monitored using TLC (silica gel/*n*-hexane/ethyl acetate/ethanol, 6:3:1); the reaction mixture was washed with cold water and the target products were purified using column chromatography (silica gel/*n*-hexane/ethyl acetate/ethanol, 6:3:1), see Table 1.

##### 2-(1-Oxociclohex-2-en[2,3-e]-2(1*H*)-oxo-3,4-dihydropiridimidin-4-yl)phenylboronic acid (**9a**)

45% yield; mp >300 °C; ^1^H NMR (DMSO-*d*_6_) δ: 8.64 (s, 1H, NH-1), 7.48 (s, 2H, OH), 7.69 (d, 1H, H-13, H-Ar), 7.52 (d, 1H, H-16, H-Ar), 7.39 (m, 2H, H-14 and H-15, H-Ar), 6.93 (d, 1H, NH-3), 6.48 (d, 1H, H-4), 2.27-1.22 (m, 6H, H-8-10); NMR (DMSO-*d*_6_) δ: 160.0 (C-2 and 7, C=O), 158.1 (C-6, C=C), 155.6 (C-11, C-Ar), C-12 no observed, carbon *ipso* to boron, 153.7 (C-5, C=C), 131.0 (C-13, C-Ar), 130.2 (C-16, C-Ar), 128.4 (C-14, C-Ar), 122.4 (C-15, C-Ar), 37.5 (C-8, CH), C-4 no observed (CH), 29.0 (C-10, CH_2_), 21.0 (C-9, CH_2_); ^11^B (DMSO-*d*_6_) δ: 29.26 as trivalent boron and 1.18 as tetravalent boron; IR (KBr) cm^−1^: 3446 (OH), 1680 (C=O), 1624 (C=C), 1390 (B-O), 1050 (B-C); FAB^+^*m*/*z* (% ra) (6 KeV): 395(1) ion-matrix interaction [M + H + Matrix-interaction]^+^, 217(2) [M − 69]^+^, 165(3) [M − 121]^+^; FAB^+^-HRMS: Molecular matrix interaction ion, for C_17_H_24_^11^B_1_N_2_O_6_S_1_, observed 395.1443 Da/estimated 395.1448 Da, for C_12_H_13_N_2_O_2_, observed 217.0971 Da/estimated 217.0977 Da, for C_8_H_9_N_2_O_2_, observed 165.0658 Da/estimated 165.0664 Da; isotopic contribution (^1^B) *versus* the loss of hydrogen, for C_17_H_24_^1^B_1_N_2_O_6_S_1_, observed 394.1487 Da/estimated 394.1484 Da, for C_17_H_23_^11^B_1_N_2_O_6_S_1_, observed 394.1364 Da/estimated 394.1370 Da.

##### 3-(1-Oxociclohex-2-en[2,3-e]-2(1*H*)-oxo-3,4-dihydropiridimidin-4-yl)phenylboronic acid (**9b**)

49% yield; mp >300 °C; ^1^H NMR (DMSO-*d*_6_) δ: 8.79 (s, 1H, NH-1), 8.57 (s, 3H, H-12, H-Ar and OH), 8.31 (s, 1H, NH-3), 8.08 (d, 1H, H-14, H-Ar), 7.92 (d, 1H, H-16, H-Ar), 7.56 (t, 1H, H-15, H-Ar), 5.43 (s, 1H, H-4), 2.24–1.74 (m, 6H, H-8-10); ^13^C NMR (DMSO-*d*_6_) δ: 193.5 (C-2 and 7, C=O), 155.4 (C-6, C=C and C-12, C-Ar), C-13 no observed, carbon *ipso* to boron, 140.1 (C-5, C=C), 135.5 (C-11, C-Ar), 130.7 (C-14, C-Ar), 128.2 (C-16, C-Ar), 115.8 (C-15, C-Ar), 36.4 (C-4, CH), 33.5 (C-8, CH_2_), 28.9 (C-10, CH_2_), 20.8 (C-9, CH_2_); ^11^B (DMSO-*d*_6_) δ: 32.89 as trivalent boron; IR (KBr) cm^−1^: 3447 (OH), 1681 (C=O), 1626 (C=C), 1372 (B-O), 1062 (B-C); FAB^+^*m*/*z* (% ra) (6KeV): 395(11) ion-matrix interaction [M + H + Matrix-interaction]^+^, 217(14) [M − 69]^+^, 165(8) [M − 121]^+^; FAB^+^-HRMS: Molecular matrix interaction ion, for C_17_H_24_^11^B_1_N_2_O_6_S_1_, observed 395.1443 Da/estimated 395.1448 Da, for C_12_H_13_N_2_O_2_, observed 217.0971 Da/estimated 217.0977 Da, for C_8_H_9_N_2_O_2_, observed 165.0658 Da estimated 165.0664 Da; isotopic contribution (^1^B) and the loss of hydrogen, for C_17_H_24_^1^B_1_N_2_O_6_S_1_, observed 394.1487 Da/estimated 394.1484 Da, for C_17_H_23_^11^B_1_N_2_O_6_S_1_, observed 394.1364 Da/estimated 394.1370 Da.

##### 4-(1-Oxociclohex-2-en[2,3-e]-2(1*H*)-oxo-3,4-dihydropiridimidin-4-yl)phenylboronic acid (**9c**)

18% yield; mp >300 °C; ^1^H NMR (DMSO-*d*_6_) δ: 8.27 (s, 1H, NH-1), 7.74 (d, 1H, H-13 and 15, H-Ar), 7.57 (d, 1H, NH-3), 7.28 (d, 2H, H-12 and 16, H-Ar), 7.21 (s, 2H, OH), 5.87 (s, 1H, H-4), 2.12–1.71 (m, 6H, H-8-10); ^13^C NMR (DMSO-*d*_6_) δ: 176.4 (C-2 and 7, C=O), 154.6 (C-6, C=C), 147.4 (C-11, C-Ar), C-14 no observed, carbon *ipso* to boron, 134.3 (C-15, C-Ar), 133.3 (C-13, C-Ar), 127.3 (C-5, C=C), 126.0 (C-16, C-Ar), 125.3 (C-12, C-Ar), 36.4 (C-4, CH), 33.6 (C-8, CH_2_), 24.6 (C-10, CH_2_), 21.0 (C-9, CH_2_); ^11^B (DMSO-*d*_6_) δ: 38.92 as trivalent boron; IR (KBr) cm^−1^: 3414 (OH), 1668 (C=O), 1613 (C=C), 1368 (B-O), 1021 (B-C); FAB^+^*m*/*z* (% ra) (6KeV): 395(6) ion-matrix interaction [M + H + Matrix]^+^, 217(9) [M − 69]^+^, 165(9) [M − 121]^+^; FAB^+^-HRMS: Molecular matrix interaction ion, for C_17_H_24_^11^B_1_N_2_O_6_S_1_, observed 395.1443 Da/estimated 395.1448 Da, for C_12_H_13_N_2_O_2_, observed 217.0971 Da/estimated 217.0977 Da, for C_8_H_9_N_2_O_2_, observed 165.0658 Da/estimated 165.0664 Da; isotopic contribution (^1^B) *versus* the loss of hydrogen, for C_17_H_24_^1^B_1_N_2_O_6_S_1_, observed 394.1487 Da/estimated 394.1484 Da, for C_17_H_23_^11^B_1_N_2_O_6_S_1_, observed 394.1364 Da/estimated 394.1370 Da.

## 4. Conclusions

The one-pot green production of 11 novel hybrid BCCs, *i.e.*, eight 1,4-dihydropyridines and three 3,4-dihydropyrimidinones was achieved. Three different activation modes (mantle heating, microwave or near-infrared irradiation) were comparatively used, in the presence of ethanol, which is a green solvent. A green-approach is offered, and an analysis of the green chemistry protocol for the overall procedure was performed. The 11 molecules are new, so appropriate spectroscopic characterizations were performed. The molecular masses of the target products were determined using ions perceived by FAB^+^-HRMS with thioglycerol as the matrix. We are performing detailed studies of these compounds for use as vasodilators and apoptotic inducers.

## Figures and Tables

**Table 1:** Comparison of production of BCDHPy and BCDHPm esters.

Compound	Solid color	Time (h)/Temp (°C)			Yield (%) *			mp (°C)
	
		Reflux	IR	MW	Reflux	IR	MW	
5a	yellow	24/85	12/100	0.25/100	3.3	11.8	56.3	190–195
5b	yellow	24/85	12/100	0.25/100	3.2	11.7	72.5	155–158
5c	yellow	24/85	12/100	0.25/100	5.9	17.2	80.2	144–146
6a	yellow	24/85	12/100	0.25/100	4.3	13.4	31.5	156–160
6b	yellow	24/85	12/100	0.25/100	6.3	16.8	72.1	198–201
6c	yellow	24/85	12/100	0.25/100	4.9	11.9	68.9	150–154
7b	brown	24/85	12/100	0.25/100	6.9	14.6	60.0	>300
7c	brown	24/85	12/100	0.25/100	5.4	13.4	43.5	>300
9a	brown	24/85	12/100	0.33/140	3.3	11.7	45.4	>300
9b	brown	24/85	12/100	0.33/140	5.0	12.2	48.9	>300
9c	brown	24/85	12/100	0.33/140	3.4	11.8	17.8	>300

MH: Mantle heating; MW: Microwave irradiation; IR: Infrared irradiation;

* Yields are of isolated pure products.
